# WHO-listed authorities (WLA) framework: transparent evidence-based approach for promoting regulatory reliance towards increased access to quality-assured medical products

**DOI:** 10.3389/fmed.2024.1467229

**Published:** 2024-09-23

**Authors:** Alireza Khadem Broojerdi, Anna Laura Salvati, Mohammed Refaat Abdelfattah, Razieh Ostad Ali Dehaghi, Hiiti B. Sillo, Rogerio Gaspar

**Affiliations:** Regulation and Prequalification Department, World Health Organization, Geneva, Switzerland

**Keywords:** WHO listed authority, WLA, medicines regulation, stringent regulatory authorities, global benchmarking tool, reliance, regulatory systems strengthening, national regulatory authority

## Abstract

**Background:**

Increased global access to safe, effective and quality-assured medical products remains a primary goal for the full realization of the World Health Assembly Resolution WHA 67.20 on regulatory systems strengthening for medical products as well as target 3.8 of the Sustainable Development Goals (SDG). To promote the development of efficient regulatory systems, the WHO introduced the Global Benchmarking Tool (GBT) in 2016, upon which the WHO–Listed Authority (WLA) framework was later established. This study aimed to appraise the development of the WLA framework across various phases while highlighting its achievements, challenges, and areas for improvement.

**Methods:**

An exploratory study design using a qualitative approach was used to gather information from relevant documents as well as views and experiences from purposefully selected participants from diverse backgrounds. Data was collected using a combination of desk reviews and In-depth one-to-one or small group interviews employing semi-structured interview guides with open-ended questions. Data was analysed using an inductive thematic analysis approach.

**Results:**

The leading role of the WHO was noted in developing and implementing essential documents and mediating consultative processes among stakeholders. The framework was revealed to bring an evidence-based, inclusive, and transparent approach to recognizing regulatory authorities (RAs) operating at the highest standards of performance. The framework was anticipated to promote regulatory reliance among all RAs, the WHO’s prequalification programme, and procurement agencies. Furthermore, remarkable progress towards WLA listing was noted among transitional WLAs including the Stringent Regulatory Authorities (SRAs). Challenges related to the availability of resources, resistance to change, and complexity were associated with the framework.

**Conclusion:**

The study provides a well-rounded view with regard to the roles of the WHO, Member States and other stakeholders in establishing and operationalizing the WLA framework. Furthermore, evaluating the performance and possible WLA designation of RAs operating at international regulatory standards underscores its high relevance in contributing to public health globally. Maintenance along with timely addressing of highlighted next steps to improve the framework particularly in creating better understanding, more communication, and coordination are highly encouraged.

## 1 Introduction

Regulatory Authorities (RAs) are increasingly challenged by the need to adapt to emerging technologies that bring forth innovative products for which very limited regulatory expertise exists (1, 2). Moreover, the rising trends of Substandard and Falsified (SF) medical products pose an imminent threat to global public health security (3).

Suboptimal and inadequately harmonized regulatory systems substantially limit the effective sharing of regulatory information, transparent approaches, and reliance on regulatory decision-making. In that, the European Union (EU), among other regions, has exemplified a successful transformation of originally divergent national regulatory systems into a well harmonized regional regulatory network which has promoted the establishment of similar undertakings in other regions. In the absence of harmonized and coordinated regulatory efforts, RAs are forced to rely on their limited capacities to discharge a broad array of regulatory functions (4–6). Further, the impacts of globalization and the expansion of global trade necessitate collective interventions to promote the advancement of regulatory systems. Such challenges, among others, have substantiated the efforts by the World Health Organization (WHO) and other stakeholders in devising more effective approaches to build regulatory capacity, harmonization, and collaboration in different forms (7–9).

The establishment of the WHO Global Benchmarking Tool (WHO-GBT) in 2016 marked a significant milestone in the WHO’s efforts to advance transparency and capacity building in regulatory practices (8, 10–12). The step came as a means of implementing the recommendations of the World Health Assembly (WHA) Resolution 67.2 in 2014. Through the GBT, WHO has managed to use independent experts in generating evidence and evaluating the RAs’ overarching regulatory framework and eight key regulatory functions (10–12). Further, the GBT has introduced the concept of categorizing RAs into Maturity Levels (MLs) as adopted from ISO 9004 (11, 12).

Since its introduction, the GBT has demonstrated extensive benefits in terms of providing a structured approach for evaluating regulatory systems, promoting Good Regulatory Practices (GRPs) principles, and enablers, as well as regulatory collaboration and reliance (13). Additionally, the tool has enabled RAs to identify their strengths and weaknesses, formulate Institutional Development Plans (IDPs), and implement suggested improvements (10, 11, 14).

Over time, with the increased use of the GBT, the achievement of Maturity Level 3 (ML3) was recognized as an essential target for a regulatory authority to be considered as applying an acceptable level of regulatory oversight (WHA 67.20). ML3 refers to the third out of four Maturity Levels on the WHO-GBT which indicates that the respective RA has a stable, well-functioning and integrated regulatory system. While working with Member States towards this objective, and to leverage the capacity of already advanced authorities to increase access to quality-assured medicines and vaccines, as well as to guide procurement decisions, WHO together with the Global Fund adopted the concept of Stringent Regulatory Authority (SRA) (11). As per the current definition, SRAs are either members and observers of the International Council for Harmonization of Technical Requirements for Pharmaceuticals for Human Use (ICH) or are RAs with legally binding agreements on mutual recognition with ICH members as before the 23*^rd^* of October 2015 (15, 16).

SRAs are stated to possess adequate regulatory resources, robust and transparent procedures, and high levels of industrialization to enable optimal discharging of all regulatory functions (Mace 2021). Since their inception, SRAs have played a big role in guiding regulatory reliance by the WHO Prequalification (PQ) programme and RAs from across many countries and regions. Moreover, procurement bodies at national, regional and international levels have been guided by Marketing Authorization (MA) granted by SRAs in procuring medical products (11, 15).

Despite notable achievements, the SRA concept faces criticism in aspects of not admitting additional members, implying the lack of harmonized stringency among other RAs, and having a skewed distribution of SRAs to the industrialized global north. Furthermore, the generalized SRA designation of all regulatory functions and product categories, the absence of a comprehensive and transparent evaluation process, and the lack of a mandate to assess regulatory capacity by the ICH are perceived to affect the credibility of the SRA concept (11, 15).

Building upon the strong foundation of the GBT, designating RAs as WHO Listed Authorities (WLA) was prompted by the requests of Member States and as it was discussed during the 17*^th^* International Conference of Drug Regulatory Authorities (ICDRA) in 2016 in South Africa. The request was further endorsed by WHO’s Expert Committee on Specifications for Pharmaceutical Products (ECSPP) (11, 17, 18). Following these events, transitional arrangements were necessary before embarking on the full operationalization of the WLA framework. Such arrangements included replacing the WHO interim list of NRAs with the transitional WLA (tWLA) list which was assigned the validity of five years starting from the publication date of the Interim WLA operational guidance. Briefly, the tWLAs comprise of RAs operating at ML3 or ML4, SRAs, NRAs of regional reference in the region of the Americas, as well as Functional or Highly performing NRAs for vaccines. These arrangements aimed at i) recognizing achievements and work of all RAs in the interim list, ii) protecting the global supply chain of quality-assured medical products, iii) offering a clear and transparent path for RAs on the list to becoming WLAs, and iv) ensuring that the processes are feasible and efficient (14, 18).

A WLA is formally defined as “A regulatory authority (RA) or a regional regulatory system (RRS) which has been documented to comply with all the relevant indicators and requirements specified by WHO for the requested scope of listing based on an established benchmarking and performance evaluation process” (13, 16, 18, 19). The framework is purposed to provide a transparent process for global recognition for RAs and RRSs operating in conformity to internationally recognized standards, guidelines, and GRPs (11, 14, 16, 20). The introduction of such values was aimed at building trust among RAs, improving regulatory systems, expanding the pool of reliable RAs, and ultimately promoting access to safe, effective and quality-assured medical products (11, 13, 16). This study focused at appraising the development of the WLA framework across various phases while highlighting its achievements, challenges, and areas for improvement.

## 2 Methodology

We employed an exploratory qualitative study design to investigate various aspects of the WLA framework by reviewing selected documents and interviewing key participants from across the WHO, WLAs, transitional WLAs, donors, pharmaceutical industries and international procurement agencies. A total of 17 documents including peer-reviewed articles from recognized scientific journals, policies, concept notes, manuals, operational guides, technical reports, and assessment tools were appraised through desk reviews (Supplementary material).

Purposeful sampling was used to obtain 14 organizations from different categories established to be important players across different phases of conceiving, developing and operationalizing the WLA framework (Table 1). Following the same sampling technique, a total of 27 participants were selected including at least one participant from each organization. Selection of the individual participants was made by either the WHO or their respective organizations based on their involvement with the WLA framework, experience, and nature of their roles.

**TABLE 1:** Summary description of recruited organizations, their categories and respective number of interviewed participants.

Category/target group	Organization−Country/Office	Number of participants
WHO Headquarters and Regional Offices	The World Health Organization (WHO)−Headquarters	4
	The World Health Organization (WHO) −South-East Asia Regional Office (SEARO)	1
	Pan American Health Organization (PAHO/AMRO) −Latin America	1
WHO Listed Authority (WLA)	Ministry of Food and Drug Safety (MFDS) −South Korea	2
	Swiss Agency for Therapeutic Products (Swiss Medic) −Switzerland	2
Transitional WHO Listed Authorities (tWLAs)	European Medicines Agency (EMA) −Europe	2
	Agency for Medicinal Products and Medical Devices (HALMED) −Croatia	1
	United States Food and Drug Administration (US-FDA) −United States	3
NRAs practicing reliance on SRAs	South African Health Products Regulatory Authority (SAHPRA) −South Africa	1
	Ghana Food and Drug Authority (Ghana FDA) −Ghana	1
Agencies involved in international procurements of health products	United Nations Development Agency (UNDP) −Headquarters	1
	United Nations Children’s Emergency Fund (UNICEF) −Headquarters	5
Donors, stakeholders and partner organizations to the WHO	Bill and Melinda Gates Foundation (BMGF) −Headquarters	1
	Council for International Organizations of Medical Sciences (CIOMS) −Headquarters	1
	International Federation of Pharmaceutical Manufacturers and Associations (IFPMA)−Headquarters	1

A combination of one-to-one and small-group in-depth interviews was carried out (between September 2023 and April 2024) based on the available number of participants from the respective organization. We used a semi-structured interview guide with open-ended questions to gather the views and experiences of the study participants regarding the historical background, objectives, benefits, challenges, and suggestions regarding the WLA framework, among other aspects (Supplementary material). A unique interview guide was used for each of the six categories of participants’ organization (Table 1). The interview guides were tested for their suitability via a combination of peer debriefing and pilot testing involving the first two participants from each category. Subsequent alterations to the tools were undertaken to enhance the clarity, flexibility, and adequacy of allocated time.

The same interviewer conducted all interviews through video calls on an online platform (Zoom Video Communications, California, US). Interviews were conducted in English language and lasted for 45 – 60 minutes. We established the saturation of obtained information upon observing the recurrence of similar themes from participants among each target group. Upon reaching this point, no further participants were recruited.

Inductive thematic analysis was employed to evaluate the obtained data as per the guidance provided by Braun and Clarke (21). The approach was selected due to having an extensive dataset, a shortage of literature on the subject matter, and the intention to ensure greater flexibility in identifying, analysing, and reporting the available themes and patterns (9, 21, 22). Following the transcription process, we performed further analyses of the data and generated the respective narratives as per the procedures outlined in our previous work (9). The selection of individual highlighted quotes from the participants was based on the virtue of providing the best representation of the respective theme, offering unique insights, special emphasis, diversity of perspectives, as well as effective communication of the points.

Each study participant was provided with detailed informed consent and voluntarily took part in the study. To avoid bias and ensure confidentiality, the organizations’ and participants’ identities were concealed during the first transcription and replaced by codified identifications.

## 3 Results

### 3.1 Role of the WHO towards operationalization of the WLA framework

The role of the WHO through its different units and teams was recognized across the major areas of providing leadership as well as coordinating collaborative efforts and communication. The study has found crucial roles of the WHO in the development and implementation of policies, guidance documents, and the Performance Evaluation (PE) framework (Table 2). The WHO was also acknowledged in the initiation and overseeing of consultative processes through engaging with the public, experts from Member States, relevant WHO teams, regional offices, and all key stakeholders such as funders and global procurement agencies.

**TABLE 2:** Overview of aspects covered in different documents issued by the WHO with respect to the WLA framework.

Covered Domains/Aspects	WHO Global Benchmarking Tool (12)	WLA Concept note (11)	WLA Policy Document (16)	PE Manual (23)	WLA Operational guidance (14)	TAG-WLA Terms of Reference (20)	WHO TRS No. 1033 (13)
Historical background on the WLA framework		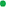	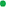		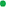	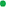	
SRA concept and/or WHO-Prequalification programme		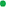	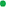		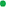	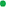	
GBT based WHO maturity levels	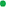	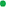	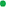	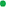			
Roles of the WHO and stakeholders		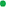	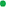		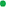	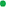	
Objectives and description of the WLA framework		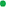	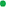		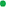	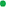	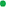
Criteria and progress for WLA listing		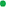	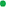	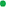	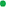		
WLA and objectives of the Resolution WHA 67.2	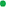	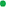	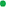		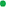		
Status achieved by RAs towards WLA listing					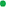		
Impact of WLAs on regulatory outputs, outcomes, and impact		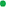	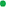		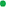	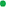	
Impact of WLA in regulatory collaboration, convergence, harmonization, and reliance		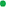			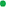	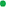	

PE, Performance Evaluation; TAG, Technical Advisory Group; TRS, Technical Report Series; WLA, WHO Listed Authority; full details of the documents are provided

### 3.2 Benefits of the WLA framework

In the context of regulatory systems, the WLA framework was regarded by many participants as bringing forth an evidence-based, objective, and transparent approach to recognizing RAs operating at high standards. Compared to the SRA concept, the framework was anticipated to offer greater flexibility by allowing the listing of one or more of the WHO-recommended regulatory functions, and product categories, as well as facilitating a more equitable geographical distribution of WLAs.

*“I must say that, if there is one transformative concept that WHO has introduced over time that will have an impact on regulatory oversight over products, it is this WLA framework.” (Participant 2, WHO Headquarters).*

*“The difference here is this (WLA) is evidence-based, that an assessment is done and there is a minimum set of standards that all the WLAs meet, and I think that’s highly beneficial…, another huge benefit is that countries may feel much more confident, relying on the work of a regulatory authority within their region.” (Participant 15, tWLA 2-SRA).*

Furthermore, the framework was commended for promoting investment in regulatory systems, along with fostering regulatory collaboration, convergence, good reliance practices and good regulatory practices by yielding higher trust in agencies with proven levels of good performance beyond the assessment of the configuration of the regulatory system. These values were viewed to optimize resources and highly support the WHO-PQ programme in expanding the pool of reliable experts, regulatory authorities, and product types. Additionally, the established transitional arrangements were regarded as giving adequate time to RAs and other stakeholders who rely on SRAs to update their respective policies, laws, and guidelines. This is in line with the creation of three possible pathways (standard, abridged, and streamlined) for RAs of different backgrounds to undergo PE based on the level of pre-existing evidence. These efforts were geared towards optimizing the use of available resources while providing robust frameworks for relying on the current and future WLAs.

*“We are expanding the pool of authorities that others can rely upon, including our own Pre-qualification program which will also rely on authorities beyond the current SRAs.” (Participant 5, WHO-Headquarters).*

*“…if you have this WLA, it is very clear, you can even put it in the law, that if we use reliance, we use it based on what the WHO has done, these are authorities you can rely on, they are trustworthy partners.” (Participant 26, Partner Organization 1).*

Moreover, the framework was anticipated to increase global access to safe, effective and quality-assured medical products, hence the promotion of public health. There was a common agreement among participants from procurement agencies regarding the potential of the framework to increase the number of reliable suppliers (due to effective regulatory oversight), streamline procurement processes, and ensure effective responses to public health emergencies. Designation of WLAs was also expected to yield economic rewards to manufacturers and governments, by facilitating equitable and timely access to global markets for products regulated by WLAs.

*“I think as industrial stakeholders we are very supportive of the WLA framework; we understand it is a good process for recognition and to have a better or a more comprehensive program for assessment.” (Participant 27, Stakeholder Organization-Manufacturing and Supply of Pharmaceuticals).*

### 3.3 Necessary resources and support for operationalization of the framework

The study has identified the allocation of adequate personnel, time, and financial resources to be essential requirements for operationalizing the framework. The current WLAs and tWLAs undergoing PE reported putting in place task forces comprised of dedicated staff with required expertise, including those from outside the RAs. Other participants pointed out the essence of effective mechanisms for planning, prioritization, quality assurance, as well as the involvement of NRA’s top management and the government throughout the PE process.

*“The main challenge was to find out who is the best possible expert or where is the best possible expertise in our agency to answer those questions and to bring the documentary evidence.” (Participant 10, WLA 2).*

Furthermore, the WLAs reported being supported by the WHO in the form of overall guidance, clarification of complex aspects, and access to information. Concerning other resources, the studied WLAs and tWLAs under PE were notably self-sufficient in facilitating the listing process.

### 3.4 Clarity regarding the WLA framework

**FIGURE 1:**
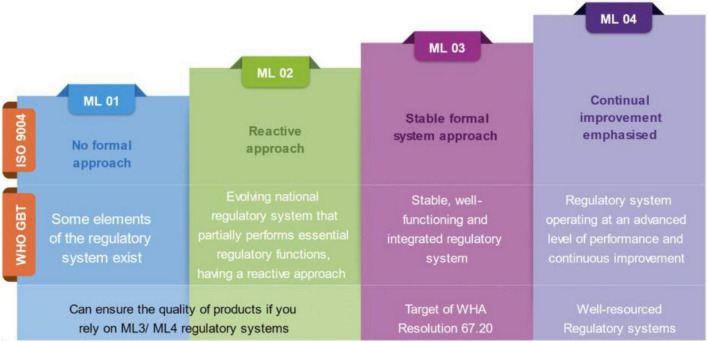
Description of the meaning and expectations of the four Maturity Levels (ML) as per the original ISO 9004 categorization and its subsequent adoption under the WHO-Global Benchmarking Tool (GBT).

The lack of detailed understanding of the WLA framework was a commonly inferred challenge among participants of diverse backgrounds. This was mostly revealed by information gaps regarding the objectives of the framework and its difference from the GBT-based maturity levels with respect to reliance and guiding procurement activities (Figure 1).

*“…but if we were to ask the difference between Maturity Level 3 and 4, and WLA, I don’t think it is so clear and, beyond that, I think in terms of reliance they are also not very clear… I’m not yet convinced that there is sufficient clarity on how WLAs can contribute to establishing reliance mechanisms and how this can impact the procurement dimension.” (Participant 4, WHO Regional Office).*

Furthermore, participants from the WHO and partners underlined the need for the GBT to be understood as a capacity-building instrument, whereas the performance evaluation for the WLAs was designed to measure the performance of regulatory systems, and the WLA framework was thus conceptualized to promote regulatory reliance at different levels.

*“GBT was never designed to be used for performance assessment or for establishing reliance mechanisms to be used in a procurement setting. That was never the case. We need to be very clear on this aspect and to distinguish between the two approaches: GBT is for capacity building, it is not for the procurement, it is not for the performance assessment, that is WLA” (Participant 1, WHO Headquarters).*

*“I believe there is a difference between possessing a specific regulatory capacity at a given point in time and achieving the necessary level of performance.” (Participant 25, Donor and stakeholder organization in the supply chain of medical products).*

### 3.5 Acceptability and progress towards WLA listing

The WLA framework was found to be highly acceptable among participants from diverse backgrounds. The expression of interest by multiple SRAs to become WLAs was regarded as an essential factor in ensuring a smooth transition between the two concepts. High levels of confidence, determination, and commitment were noted among participants from the tWLAs regarding the attainability of the WLA listing in the given timeframe of five years. Apart from the good progress among tWLAs to undergo PE, some participants from the WHO expressed uncertainties about the timely completion of the transition by some tWLAs. The delay was perceived to originate from differences in priorities, levels of commitment and availability of resources.

### 3.6 Complexity of the performance evaluation process

Resistance to change due to the strong desire to maintain the status quo, fear of the unknown, and concerns about potential disruption of the global medicines supply chain were experienced from within and outside of the WHO. Further, participants from tWLAs and WLAs pointed out difficulties in striking a balance between transparency and confidentiality along the listing process. This is because the WLA framework strongly promotes transparency in all regulatory activities, a configuration that has created problems in some jurisdictions where legal constraints on confidentiality issues are extremely challenging. Nonetheless, such requirements are cross-cutting, hence necessitating all candidate WLAs to be ready to abide by them.

Hurdles in managing priorities between undergoing PE and discharging routine regulatory functions, as well as complexities in securing inputs from multiple centers or departments within the NRAs, were also stated. Securing input from all players within the organization was notably necessitated by the nature of PE to request in-depth details of all regulatory functions.

*“I think it is a very heavy process and I understand why it is heavy and complicated.” (Participant 11, tWLA 1).*

In addition to the newness and complexity of involved processes, which were perceived to be comprehensive, difficulties in interpreting and understanding the language and requirements of different PE indicators were shared among participants. Furthermore, participants from the WHO reported facing challenges in aligning diversity related to regional differences, legal and policy issues, and avoiding the negative influence of political imperatives on technical aspects of the framework.

*“On the political side, especially with the impetus for local production, both politicians and manufacturers are seeing that their national regulatory authorities should become WLAs now, for them to be able to participate in global trade, and they are often applying a lot of pressure to the WHO, and those pressures are a big challenge.” (Participant 2, WHO-Headquarters).*

### 3.7 Modular approach and scope of WLA listing

The modular approach of the WLA framework allows for the stepwise listing of specific regulatory functions or product categories. This approach was requested by Member States to ensure more flexibility in attaining the WLA designation. Nevertheless, this was perceived to bring complexity and confusion to some participants from international procurement agencies. The participants anticipated laborious and lengthy screening of the listed WLAs before arriving on procurement or other reliance decisions.

*“…but for me, the biggest challenge is that they are listed for specific functions, that makes it challenging for the end user (e.g. procurement agencies) to keep track of what functions the WLAs are listed for.” (Participant 22, UN Agency involved in global procurements-1).*

However, other participants expressed opposing views in favor of functions and product category-based listing of WLAs.

*“…this complexity is a challenge, but I think there is no easy way around it, because this way (listing of specific functions and product categories) of approaching the framework was demanded by Member States.” (Participant 2, WHO-Headquarters).*

*“WLAs should be linked to certain product categories because it is impossible to say that any small agency can power equally well everything, it is just not realistic, this is not happening.” (Participant 26, Partner Organization 1).*

Moreover, the need for the inclusion of medical devices within the scope of the WLA framework was commonly shared among participants from global procurement agencies, being perceived as an urgent and necessary future development of the framework to guarantee patients’ access to a broader range of medical products.

## 4 Discussion

### 4.1 Roles of the WHO and Stakeholders

The WHO has played vital roles across different phases of developing and implementing the WLA framework. This is demonstrated by a widespread recognition and appraisal of its roles among participants of diverse backgrounds as well as a set of documents that form strong pillars of the entire WLA framework (12, 14, 16, 23). Nevertheless, that success would not have been possible without the notable support from Member States, stakeholders, partner organizations, donors, and the public at large. Furthermore, the respective interdependencies between the WHO, ICDRA and ICH in the creation of overarching health policies, facilitation of dialogues and cooperation, and development of technical guidelines for the regulation of medical products and harmonization are extremely valuable in ensuring a unified approach to enhancing the quality, safety, and efficacy of pharmaceutical products globally. These findings underscore the essence of effective leadership, coordination, and documented guidance in executing complex and multifaceted programmes involving diverse players (23–25).

### 4.2 Realized benefits of the WLA framework

The concrete outcomes of the WLA framework include bringing forth a significant transformation in advancing regulatory outputs, outcomes and impact, ultimately contributing to the promotion of public health globally. Based on the demonstrated higher level of transparency and evidence-based listing of WLAs, the framework is on the right course to the full realization of its objectives including offering an outstanding contribution to the development of good reliance practices. Contrary to the SRA concept, the expected increase in the number of WLAs over time guarantees their broader global distribution, hence providing a closer collaborating hand to an increasing number of NRAs (11, 16). However, for effective realization of such benefits, countries, regional and global entities must put in place enabling environments for smooth collaboration and reliance on WLAs. To this end, changes in the global regulatory landscape due to the introduction of the WLA framework necessitate parallel efforts among all Member States and stakeholders to align with its objectives, processes, and implications (26, 27).

### 4.3 Resource allocation and technical support

Resources of varying nature constitute a critical aspect for the operationalization of the framework on the side of the WHO as well as the RAs. This study has highlighted the need for careful evaluation, planning, and allocation of needed assets before undergoing the PE process for WLA listing. Considering inequalities among countries, there is a strong need for support mechanisms to ensure that the prospect for WLA listing is open for all RAs desiring to be listed based on self-evaluation (4). Such measures should include encouraging countries to prioritize budgeting for strengthening regulatory capacity, increased investment in staff training, and seeking financial and technical support from governments and external stakeholders (4, 9).

### 4.4 Clarity, acceptability and progress towards WLA listing

Regardless of broad acceptability, and aspirations for achieving the status, information gaps still exist, and some stakeholders are still confusing the purposes of the WLA framework to that of the GBT. Furthermore, there are concerns about the timely achievement of the transition to fully listed WLA status among tWLAs as well as the limited level of clarity and understanding of the WLA framework due to its newness or being newer compared to the GBT modality. The ascending nature of levels in the GBT framework has led to the general perception of ML4 as the highest and hence most competent NRAs even in terms of performance. However, this is not the case as the GBT is not designed for thoroughly measuring the performance of RAs (12, 23). To facilitate smooth transitioning and draw maximum benefits from the framework, the WHO, Member States, and relevant stakeholders should ensure sustainable advocacy for the framework particularly among RAs.

### 4.5 Complexity of the framework and resistance to change

Based on the perspective and routine operations of the involved party, resistance to change, process complexity, and optional listing of regulatory functions and product categories, are among the core hurdles associated with the WLA framework. Other studies have reported on resistance at individual and organizational levels following the introduction of substantial changes to the existing structures and/or operations (7, 9). However, due to the increasing number and extent of challenges related to regulatory oversight, constant improvement of the existing systems is imperative. Although there should be room for addressing difficulties related to process complexity, those undertakings should not be at the expense of the achieved framework’s robustness, transparency, and meticulous nature of the framework.

As pointed out by participants from the WHO, the current design of the WLA framework is an outcome of extensive consultative processes involving Member States and a wide array of stakeholders (14, 16). Thus, the challenges still existing in the WLA framework are mostly associated to the introduction of a new process which involves multiple and diversified stakeholders, as well as to the intrinsic comprehensiveness of the WLA framework.

Taken together, the framework’s complexity is in tandem with ensuring that it is highly trustable and credible hence contributing to the overall acceptability of the WLA concept across a wide range of stakeholders. This is confirmed by the positive attestations from the RAs which have achieved the WLA status on the extent to which the PE process has contributed to improving their regulatory outputs and outcomes. Furthermore, the current performance evaluation was reported to be lesser complex as compared to the initially proposed version. The adopted simplifications were made following public consultations and piloting in three countries and were meant to make the framework more accessible, affordable, reasonable and realistically applicable.

In recognition of the merits of the WLA initiative, collective efforts are needed to address the existing hurdles while preserving its core values (7, 15). Impressively, the WHO indicated to be taking necessary measures to provide targeted training, clarifications, and answers to specific requests, to help the stakeholders in understanding and navigating the stated complexities.

### 4.6 Recommended future steps in operationalizing the WLA framework

A number of key recommendations were discussed by the participants for further improvement and sustainability of the WLA framework. The suggested actions cut across the need for continued engagement, improved communication, and expanding the scope of products categories, among others. Table 3 provides an overview of the recommended measures.

**TABLE 3:** Summary of recommended actions towards improving and sustaining the WLA framework.

Recommendation	Details
Continued WHO’s engagement and support	• Continue to engage with and provide customized support to RAs interested in WLA listing • WHO should use existing WLAs to promote regulatory excellence through experience and expertise sharing.
Improve clarity and awareness of the WLA framework	• Enhance communication of the BGT as a capacity-building instrument. • Communicate the WLA framework as a path to evaluate and recognize regulatory performance over time
Smooth Management of the transition process	• Ensure that the transition process is as smooth as possible to avoid disruption in global supply chain of medical products • Prioritize assessments based on factors such as the applicant’s regulatory capacity, geographical distribution, and manufacturing capacity.
Develop a searchable database for WLAs	• Allow easier navigation and tracking of WLA-listed functions, product categories and geographical locations by creating a searchable and openly accessible database
Expand scope of the WLA framework	• Ensure the frameworks relevance in the changing regulatory environment by including other product groups such as medical devices, in vitro diagnostics, blood products and vector control products.
Ensure continuous improvement and monitoring	• Implement continuous improvement measures. • Establish constant monitoring of the framework’s performance through feedback mechanisms, stakeholder involvement, and dedicated impact studies after three years of operationalization
Enhance transparency, information sharing and a balance with confidentiality	• Make PE outcomes and assessment reports publicly available so as to promote trust, accountability, and knowledge sharing in regulatory decision making. • Ensure a balance between transparency/information sharing and confidentiality aspects

To this end, it is crucial that all stakeholders fully understand the intended applications of the framework and their specific roles within it. This includes regarding the WLA designation as not as a once-off event, but rather as a dynamic process involving continuous monitoring and transparent interactions, self-evaluations and collaborative efforts towards the common goal of protecting public health.

## 5 Conclusion

The study has highlighted key aspects of the WLA framework. Significant roles played by the WHO and its stakeholders, including the commitment and investment from Member States were crucial across different phases of developing the framework towards its operationalization. Moreover, through designating RAs operating at international regulatory standards, the framework will largely contribute to the advancement of regulatory outputs and outcomes, and ultimately achieve a greater and more widespread impact on global public health. Besides common acceptability, the framework is faced with several challenges including being resource-intensive, resistance to change, and lack of clarity among stakeholders. The study has put forward recommended steps to address the existing challenges to ensure smoother operationalization and full realization of the framework’s potential.

## Data Availability

The raw data supporting the conclusions of this article will be made available by the authors, without undue reservation.
