# Supplementary material for: WHO-listed authorities (WLA) framework: transparent evidence-based approach for promoting regulatory reliance towards increased access to quality-assured medical products
Source: Front Med (Lausanne). 2024 Sep 23;11:1467229. doi: 10.3389/fmed.2024.1467229 (PMC11456560; doi:10.3389/fmed.2024.1467229)
Supplement: Supplementary file 1 [file Table_1.DOCX]

**Letter of Invitation**

Dear Colleague,

Greetings,

The Regulatory Systems Strengthening (RSS) team of the World Health Organization (WHO) is conducting a study titled: “**WHO-Listed Authorities (WLA) Framework: Conception, Inception, and Progression towards transforming Global Regulatory Convergence, Harmonization and Reliance for increased access to quality-assured medical products**”.

The main goal of the study is to evaluate key aspects across the conception, inception, and progress in the implementation of the WLA framework. Furthermore, the study aims at providing a more comprehensive understanding on the challenges and opportunities associated with the operationalization of the framework.

To take part in the study, we will ask you to join in an online small group discussion or one-on-one interview meeting which will last for about 1 hour. All your personal information and responses will be anonymous and will be handled with high confidentiality. Your participation in this study will be completely voluntary and you may decide to discontinue taking part in the study at any time. Your participation is very important in enabling knowledge generation in line with the objectives of this study.

Your consent to participate in this study can only be given through reading and signing the attached consent request letter.

Thank you in advance for your kind participation,

**Dr. Alireza Khadem**

Team Lead

Regulatory Systems Strengthening

The World Health Organization, Geneva.

**Consent Requesting Letter**

Dear Colleague,

Greetings!

We are kindly requesting for your consent to participate in the study titled “**WHO-Listed Authorities (WLA) Framework: Conception, Inception, and Progression towards transforming Global Regulatory Convergence, Harmonization and Reliance for increased access to quality-assured medical products**”

This consent requesting letter is aimed at allowing you to understand about the study before deciding on participating or not to.

**The objectives of this study are:**

1. Generating and documenting a comprehensive background of the WLA framework in relation to the superseded concepts and frameworks.
2. Appraising the roles and experiences of the WHO and related parties in from the conception to the operationalization of the WLA framework.
3. Evaluating the links between the WLA initiative and the objectives of the resolution WHA 67.20
4. Providing a comprehensive description of the WLA framework and the updated status achieved by different regulatory authorities.
5. Profiling the expected influence of the WLA framework on the regulatory performance, convergence and reliance practices.
6. Assessing the potential challenges and opportunities in transitioning from the Stringent Regulatory Authority (SRA) concept to the WLA framework.
7. Determining the challenges faced by the regulatory authorities in the transitional WLA list in their efforts to attain being WLA listed.
8. Determining the experiences and future expectations of the regulatory authorities relying on WLA listed authorities.

We anticipate that the findings from this study will inform the regulatory community around the globe on all key aspects across the conception, inception, and progress in the implementation of the WLA framework. Further, the study will provide a comprehensive understanding on challenge and opportunities associated with the operationalization of the framework.

**Procedures**

You will be required to join in an online small group discussion or one-on-one interview meeting which will take about 1 hour. The discussion will involve you and the facilitator together with 1 or 2 other participants from your/other organization(s). However, in some cases, the meeting can involve a one-on-one interview between you and the facilitator. Appointments for the meeting will be set after a general consensus by all targeted participants, and we will be committed to a high degree of flexibility.

The meeting will take place on the Microsoft Teams/Zoom platforms based on the prevailing conveniences. The entire discussion session will be recorded, and the recordings will only be used for the purpose of extracting and transcribing the data intended to be collected.

During the meeting, the facilitator will moderate the session by guiding the discussion across the themes selected to match the objectives of the study (see the objectives above). Moreover, participant(s) will be encouraged to freely share any other views/insights which they perceive to be related to the study.

**Voluntary nature of the study**

Your participation in this study is completely voluntary and your decision to take part or not will be fully respected. Moreover, you can change your decision about your participation in the study and remove your consent at any time. The WHO-RSS team will not treat you or your respective organization differently, if you opt not to take part in the study.

**Privacy**

Anonymity and confidentiality will be highly observed during this study. No personal information or responses will be used outside the stated objectives of this study or shared with any other party. However, due to the data collection method other participants of the meeting will be aware of your responses.

Moreover, all personal identifiers will not be included in the data analysis and reports to be written thereafter. To enable this, coded identifications will be used for each participant. All recordings and other raw data collected will be stored under password protected data storage devices. Video recordings will not be shared to any other party.

For the purpose of disseminating the findings to a larger audience, the findings from this study will be published in a Peer-reviewed scientific journal. The WHO-RSS team will not be obliged to share the final version of the article with you or your NRA before its publication.

**Risks and Benefits of participating in the study**

There are no foreseeable risks to your safety and wellbeing by participating in this study. Your participation is very important in enabling knowledge generation in line with the stated objectives through sharing your insights towards the advancement of good regulatory practices across the world.

**Payments**

Participation in this study is completely voluntary and no payments/reimbursements will be made to the participants.

**Contacts and questions**

In case you have any questions now or later, please feel free to contact any of the Following.

**Facilitator**

Dr. Nelson Masota

[Nelson.masota@muhas.ac.tz](mailto:Nelson.masota@muhas.ac.tz)

**Coordinator, WHO-RSS Team**

Dr. Razieh Ostad Ali Dehaghi

[ostadalidehagir@who.int](mailto:ostadalidehagir@who.int)

**Statement of Consent**

**I (your full name) ____________________________________________________ from (name of your organization and country) ______________________________________________________________________________________________________________________________________**

**do consent to participate in this study.**

Signature: ________________________ Date: ____________________________

***Please send the scanned copies of this last page of the signed consent form and the filled questionnaire to the email address:*** [nelson.masota@muhas.ac.tz](mailto:nelson.masota@muhas.ac.tz) **copying** [ostadalidehaghir@who.int](mailto:ostadalidehaghir@who.int)
