# Supplementary material for: WHO-listed authorities (WLA) framework: transparent evidence-based approach for promoting regulatory reliance towards increased access to quality-assured medical products
Source: Front Med (Lausanne). 2024 Sep 23;11:1467229. doi: 10.3389/fmed.2024.1467229 (PMC11456560; doi:10.3389/fmed.2024.1467229)
Supplement: Supplementary file 2 [file Table_2.DOCX]

**Interview guides for one-on-one interviews/Small- and Focused Group Discussions.**

**Group 1: World Health Organization staff (Headquarters and Regional Offices)**

1. What have been the major roles of the WHO through your team across different phases of bringing the WLA framework into action.
2. Could you please share on any challenges encountered during the process of bringing the WLA framework into action (*Please probe more on each mentioned challenge to establish its origin*)
3. What is your general view/feeling regarding the level of acceptability of the switch from SRA to WLA among the involved stakeholders e.g. WHO, NRAs, procurement agencies, SRAs, etc.
4. To what extent do you think the NRAs from the member states understand the difference between the concepts of Maturity levels and WLA listing?
5. In your opinion, what should be the possible measures in place to ensure that as many as possible countries in the tWLA are transitioning to the WLA list before closure of the provided time window of 5 years?
6. In your opinion, what is the current degree of willingness among countries in the tWLA to become WLA? (*Probe if they can foresee any resistances in the process)*
7. Are there any factors/aspects known to you that might limit/slow down the transition process?
8. From the currently achieved status of the WLA framework, what do you perceive to be the necessary future steps?
9. Do you have any other comments or questions?

**Group 2: World Health Organization Listed Authorities (WLAs)**

1. What were the major reasons for your NRA/country to decide to undergo the evaluation process toward WLA listing?
2. Can you describe the nature and level of efforts and resources which needed to put in place by your NRA through the process? (*Probe more to establish the types of efforts/resources invested*)
3. How would you describe the nature and levels of guidance and support provided to your NRA by your government throughout the process toward WLA listing?
4. How would you describe the nature and levels of guidance and support provided to your NRA by the WHO throughout the process towards WLA listing?
5. Apart from the WHO, are there other external institutions/organizations which provided any form of support specifically directed to the process towards WLA listing?
6. Could you please share on any challenges faced by your NRA during the evaluation process? (Probe more to see how each challenge was addressed)
7. After attaining the WLA status, what are the anticipated responsibilities, opportunities, and challenges for your NRA? (p*robe to see if there are any measures to accommodate the foreseeable opportunities and challenges*)
8. What areas or subjects you think your NRA will be interested in collaboration with WHO after being listed as a WLA?
9. Do you have any other comments or questions?

**Group 3: Transitional World Health Organisation Listed Authorities (tWLAs)**

1. How does your NRA perceive its status as a tWLA?
2. What is the level of awareness in your NRA about the WLA framework?
3. Do you see any advantage in applying for becoming WLA? Or conversely, any perceived risk that may arise from not becoming a WLA?
4. Have you started any measures/steps towards being listed as a WLA? If yes, please elaborate more on that.
5. To what extent do you think the goal of your NRA being WLA listed is attainable?
6. Can you share on any currently faced or perceived challenges which might limit the progress of your NRA towards being listed as a WLA? (*probe further on the willingness of the government and allocation of required resources*)
7. In what specific areas does your NRA need external support towards attaining the WLA designation?
8. To what extent do you think your NRA is aware of the difference between the concepts of Maturity levels and WLA listing?

**Group 4: RAs practising reliance on SRAs**

1. For how long have your NRA been recognizing or relying on works ofsSRAs/WLAs?
2. What are the major regulatory functions on which your NRA has been relying on SRA/WLA?
3. What have been the major benefits in the ongoing reliance and recognition practices?
4. What are the changes in policies and guidelines in line with recognizing and relying on the works of the recently designated WLAs versus previously relied upon SRAs. (*probe to know if some measures are ongoing*).
5. Do you have any other comments or questions?

**Group 5: Agencies involved in international procurement of Health Products**

1. What is your general view on the objectives of the WHO Listed Authority (WLA) framework?
2. Could you please share on the anticipated benefits of operationalizing the WLA framework to your organization (as an international procurement agency)?
3. Are there any foreseeable procurement-related challenges challenges/limitations associated with replacing the SRA concept with the WLA framework?
4. In your opinion, what areas WHO should carefully consider for better operationalization of the WLA framework?
5. Do you have any other comments or questions?

**Group 6: Donors, stakeholders and partner organizations to the WHO**

1. What is your general view on the objectives of the WHO Listed Authority (WLA) framework?
2. Could you please share on the anticipated benefits of operationalizing the WLA framework to your organization?
3. Are there any foreseeable challenges/limitations, in your opinion, associated with replacing the SRA concept with the WLA framework?
4. In your opinion, what areas WHO should carefully consider for better operationalization of the WLA framework?
5. Do you have any other comments or questions?
