# Supplementary material for: WHO-listed authorities (WLA) framework: transparent evidence-based approach for promoting regulatory reliance towards increased access to quality-assured medical products
Source: Front Med (Lausanne). 2024 Sep 23;11:1467229. doi: 10.3389/fmed.2024.1467229 (PMC11456560; doi:10.3389/fmed.2024.1467229)
Supplement: Supplementary file 3 [file Table_3.DOCX]

Supplementary Materials-3: List of documents used for desk review

| **SN** | **Document’s Citation** | **Category** |
| --- | --- | --- |
| 1 | WHO Listed Authority (WLA): A framework for evaluating and publicly designating regulatory authorities as WHO Listed Authorities (WLA). Geneva: World Health Organization.  <https://www.who.int/initiatives/who-listed-authority-reg-authorities> | WHO Website article/page |
| 2 | WHO Technical Report Series (TRS) Procedure for assessing the acceptability, in principle, of vaccines for purchase by United Nations agencies - referencing to Highly Performing NRAs - TRS 978 annex 6-**2013**  <https://cdn.who.int/media/docs/default-source/prequal/vaccines/trs-978-61st-report-annex-6-pq-vaccine-procedure.pdf?sfvrsn=29f0ffe_2&download=true> | WHO technical report |
| 3 | WHO. Concept note: a framework for evaluating and publicly designating regulatory authorities as WHO-Listed authorities. WHO Drug Information. **2019**;33:139-58.  <https://scholar.google.com/scholar?hl=en&as_sdt=0%2C5&q=WHO-listed+authority+%22WLA%22&btnG=> | WHO Published Article |
| 4 | Guzman J, O'Connell E, Kikule K, Hafner T. The WHO Global Benchmarking Tool: a game changer for strengthening national regulatory capacity. BMJ global health. **2020**;5:e003181.  <https://doi.org/10.1136/bmjgh-2020-003181> | Peer reviewed article |
| 5 | WHO Global Benchmarking Tool (GBT) for evaluation of national regulatory systems of medical products, revision VI. Geneva: World Health Organization; **2021**.  <https://iris.who.int/bitstream/handle/10665/341243/9789240020245-eng.pdf?sequence=1> | WHO document |
| 6 | Evaluating and publicly designating regulatory authorities as WHO listed authorities: policy document. Geneva: World Health Organization; **2021**.  <https://iris.who.int/bitstream/handle/10665/341749/9789240023444-eng.pdf?sequence=1> | WHO policy |
| 7 | WHO Expert Committee on Specifications for Pharmaceutical Preparations: Fifty‑fifth report. (WHO Technical Report Series, No. 1033). Geneva: World Health Organization; **2021**.  <https://iris.who.int/bitstream/handle/10665/340323/9789240020900-eng.pdf?sequence=1> | WHO meeting report |
| 9 | Macé C, Rägo L, Ravinetto R. How the concept of WHO-listed authorities will change international procurement policies for medicines. BMJ Global Health. **2022**;6:e008109.  <https://doi.org/10.1136/bmjgh-2021-008109> | Peer reviewed article |
| 9 | Saied, A.A., Metwally, A.A., Dhawan, M., Choudhary, O.P. and Aiash, H., Strengthening vaccines and medicines manufacturing capabilities in Africa: challenges and perspectives. EMBO molecular medicine,  **2022**;*14*, p.e16287. <https://doi.org/10.15252/emmm.202216287> | Peer reviewed article |
|  | Meeting report for the Eleventh meeting of the regional alliance of national regulatory authorities for medical products in the Western Pacific: focus on regulatory preparedness during public health emergencies. Manila, Philippines; 2022 30 August - 1 September **2022**.  <https://apps.who.int/iris/bitstream/handle/10665/366641/RS-2022-GE-13-hybrid-eng.pdf?sequence=1> | WHO meeting report |
| 10 | Technical Advisory Group on WHO Listed Authorities (TAG-WLA) Terms of Reference. Geneva: Regulatory Systems Strengthening Team-World Health Organization; **2022**.  <https://cdn.who.int/media/docs/default-source/medicines/regulatory-systems/wla/tag-wla-generic-tors-qns.pdf?sfvrsn=227ee997_1&download=true> | WHO terms of reference |
| 11 | Manual for the performance evaluation of regulatory authorities seeking the designation as WHO-listed authorities, version 2.0. Geneva: World Health Organization; **2023**.  <https://iris.who.int/bitstream/handle/10665/374058/9789240076969-eng.pdf?sequence=1> | WHO manual |
| 12 | Operational guidance for evaluating and publicly designating regulatory authorities as WHO-listed authorities. Geneva: World Health Organization; **2023**.  <https://iris.who.int/bitstream/handle/10665/374054/9789240074767-eng.pdf?sequence=1> | WHO operational guidance |
| 13 | Landmark listing of first three countries as WHO-Listed regulatory Authorities, **2023**.  <https://www.who.int/news/item/31-10-2023-landmark-listing-of-first-three-countries-as-who-listed-regulatory-authorities> | WHO website article/page |
| 14 | List of National Regulatory Authorities Operating at Maturity Level 3 (ML3) and Maturity lvel 4 (ML4) (As of 13 June **2024**)  <https://cdn.who.int/media/docs/default-source/medicines/regulatory-systems/wla/list-of-nras-operating-at-ml3-and-ml4.pdf?sfvrsn=ee93064f_18&download=true> | WHO listing |
| 15 | List of transitional WLAs (tWLA) (as of May **2024**)  <https://cdn.who.int/media/docs/default-source/medicines/regulatory-systems/wla/list-of-transitional-wlas.pdf?sfvrsn=a3011e1e_16&download=true> | WHO listing |
| 16 | List of WHO Listed Authorities WLAs, **2024**  <https://cdn.who.int/media/docs/default-source/medicines/regulatory-systems/wla/list_of_wla_may24.pdf?sfvrsn=1f6c2140_36&download=true> | WHO listing |
